# Comparison of the oncological and functional outcomes among patients with high‐risk upper tract urothelial cancer undergoing segmental ureterectomy based on tumour location

**DOI:** 10.1002/bco2.70046

**Published:** 2025-06-19

**Authors:** Maksym Pikul, Prokip Gordiychuk, Eduard Stakhovsky

**Affiliations:** ^1^ Department of Plastic and Reconstructive Oncourology National Cancer Institute of Ukraine Kyiv Ukraine; ^2^ Department of Oncology Shupyk National Healthcare University of Ukraine Kyiv Ukraine

**Keywords:** upper tract, urothelial cancerorgan‐sparing surgery, radical Nephroureterectomy, urothelial cancer

## Abstract

**Introduction:**

Segmental ureterectomy (SU) represents a viable alternative to radical nephroureterectomy (RNU) for the management of distal ureteral tumours when technically feasible. However, SU of the proximal two‐thirds of the ureter is associated with higher failure rates compared to distal ureteral tumours. This study aims to compare oncologic outcomes and renal function in patients undergoing SU for tumours located in the distal versus proximal ureter.

**Methods:**

Prospective, non‐randomized cohort study, which included adult patients with high‐risk cT2–3 cN0–1 M0 ureteral tumours deemed suitable for SU, with preoperative affected kidney function > 15 ml/min. Patients were treated at a reference centre between March 2019 and March 2023. Patients were divided into two cohorts based on the primary tumour location: distal or proximal two‐thirds of the ureter. All patients received neoadjuvant chemotherapy (Gem‐Cis) and cases that underwent RNU were excluded from the study. Kaplan–Meier analysis was employed to evaluate local‐ recurrence‐free survival (L‐RFS), progression‐free survival (PFS) and overall survival (OS).

**Results:**

A total of 41 patients underwent SU (21/20 proximal/distal location). The cohorts were matched by age, sex, BMI, ECOG status, T‐stage, pN status, tumour length, preoperative eGFR, primary pathology and positive cytology (p > 0.05). Following segmental ureterectomy, all patients with distal ureteral tumours underwent neostomy reconstruction. In the proximal ureter group, reconstruction techniques included end‐to‐end anastomosis in 9 (43%), Andersen‐Heinz plasty in 8 (38%) and ureter‐ileum interposition in 4 cases (19%).

No statistically significant differences were observed between the two cohorts in terms of surgery duration, average blood loss, Grade ≥3 complications, length of postoperative stay or 30‐day readmission rate (p > 0.05). Postoperative eGFR was similar between the groups (60.4 ± 8.5 vs. 59.4 ± 11.4; p = 0.81). Furthermore, no significant differences were found between patients with proximal versus distal ureteral tumours in terms of 2‐year L‐RFS (72% vs. 85%; p = 0.29), PFS (85% vs. 77%; p = 0.69) or OS (65% vs. 77%; p = 0.43).

**Conclusion:**

The current study demonstrates that segmental ureterectomy provides comparable oncologic outcomes and renal function preservation for both proximal and distal ureteral cancer. SU can be considered a safe and effective kidney‐sparing alternative to radical nephroureterectomy in high‐risk cases, regardless of tumour location.

## 1 INTRODUCTION

Radical nephroureterectomy (RNU) has since long been considered the gold standard of care to treat localized and locally‐advanced upper tract urothelial cancer (UTUC).^1^, ^2^ The existing surgical technique proves to be a favourable choice for surgeons, marked by a commendable safety profile and minimal local recurrence rates.^3^ However, it is still unclear whether this approach is optimal for all the patients.

Data from a large series states that around 31% of cases that undergo RNUE might require systemic treatment due to locally advanced disease, local recurrence or cancer progression.^3^ Different treatment modalities were described, favouring combined treatment using surgery and chemotherapy.^4^, ^5^, ^6^ Based on gained experience, there is a significant likelihood of interrupting systemic therapy before completing the prescribed full course, which impacts oncological outcomes. The most common limitation is the deterioration of renal function, impacting the ability to deliver the entire standard treatment volume.

Upper tract urothelial cancer is highly associated with chronic kidney disease. At the moment of primary diagnosis, 51% of UTUC patients already have compromised renal function, whereas it further deteriorates after RNUE, with only 19% able to receive proper systemic chemotherapy afterwards.^7^, ^8^ One of the potential solutions is to provide patients with neoadjuvant chemotherapy before the kidney is removed. This approach seeks to initiate systemic treatment early, targeting micrometastatic disease, with the goal of reducing tumour complexity and enhancing the feasibility of subsequent surgical interventions. An alternative option involves utilizing innovative immune agents that exert minimal impact on glomerular filtration, despite the challenges posed by limited data from clinical trials mainly devoted to bladder cancer.^9^, ^10^

Certain success was received during endoscopic organ‐sparing treatment or chemo‐ablation for low‐risk disease ‐ preserving the kidney and having no influence on overall survival.^11^, ^12^, ^13^ Nevertheless, showing unfavourable results for high‐risk cohort. Segmental ureterectomy with subsequent neostomy has demonstrated that carefully selected high‐grade distal ureteral lesions can be effectively managed with organ‐sparing surgery, leading to favourable clinical outcomes.^12^ Other locations are widely considered to be more surgically complex, bearing high complication levels with unknown oncological outcomes, thus can be suggested only for low‐risk tumours in cases when kidney function is crucial.^14^, ^15^ The rationale for such separation may stem from various factors, including distinctions in anatomical zones, the potential for multifocality of the cancer or challenges in further management and follow‐up after resecting proximal ureteral tumours.

The current study **aimed** to gain insight into whether there exist particular clinical or pathological features that influence oncological outcomes in patients undergoing segmental ureterectomy of distal and proximal ureter.

## 2 MATERIALS AND METHODS

### 2.1 Study design

Current analysis was performed as a part of the PhD scientific work “Optimization of the surgical treatment of the upper tract urothelial cancer” performed in the Department of Plastic and Reconstructive Oncourology of the National Cancer Institute and Department of Oncology of the Shupyk National Healthcare University of Ukraine. The non‐randomized prospective study analysing efficacy of the combined organ‐sparing surgical strategy in UTUC was started in March‐2019 and ended recruitment in March‐2023.

Inclusion criteria for the organ‐sparing approach were:

- tumour size ≥ 2 cm
- high‐grade urothelial cancer suspected on biopsy/cytology or both
- unifocal lesion
- no distant metastasis present
- absence of carcinoma in situ
- no evidence of concomitant UTUC or bilateral disease
- clinical N0 or N1 (lymph nodes enlarged 2 cm or less in the greatest dimension)
- preserved kidney function on the affected side with more than 15 ml/min GFR; unaffected contralateral kidney with no signs of obstruction
- ECOG 0–2; ASA I‐III scoring

Present article aimed to evaluate subgroup data from the patients that underwent SU. Cases were split into 2 cohorts depending on the primary tumour location: distal (lower 10 cm from the bladder) and proximal (upper 15–20 cm starting from pyelo‐ureteral segment) ureter. Outcomes resulting in the primary radical nephroureterectomy (RNUE) were excluded from the current analysis.

The study was approved by the Institutional Review Board and the local ethics committee (local ethics committee agreement № 134, Kyiv, 26.03.2019;) and was conducted according to the Declaration of Helsinki and the Good Clinical Practice guidelines. The databases used in the study are the intellectual property of the National Cancer Institute of Ukraine. A special informed consent was designed for the study and signed by all the patients in the presence of the urologists involved in the investigation.

### 2.2 Sample size

A minimum of 20 patients per cohort was planned prior to study initiation, based on estimated recruitment feasibility. Although no formal sample size calculation was performed, potential reasons for dropout—such as loss to follow‐up, patient withdrawal of consent, deterioration of general condition prior to surgery or unforeseen treatment delays—were considered during the planning phase. To minimize these risks, strict inclusion criteria were applied, and a structured follow‐up schedule was maintained in close coordination with physicians from the outpatient clinics.

### 2.3 Primary management of upper urinary tract obstruction

The key target of primary treatment was to eliminate upper tract obstruction where it was present. In cases, when it was not removed by ureteral stenting or nephrostomy in the referring hospital, it was performed as the first treatment step. Upper tract decompression secured the kidney from further function deterioration and recurrent inflammation. In cases where placing a double J stent was impossible due to ureteral narrowing or if the stent did not remove the obstruction, a nephrostomy procedure was performed. Antibacterial therapy to treat urinary tract inflammation was performed according to the guidelines before the start of the main treatment course.

### 2.4 Diagnostic evaluation

To obtain data about expected surgical complexity contrast‐enhanced Computed Tomography (CT) scan with excretory phase was performed. Kidney function was evaluated using creatinine and estimated glomerular filtration rate (eGFR). Additionally, all patients included in the study underwent dynamic renal scintigraphy; in cases with primary hydronephrosis, it was performed after deobstruction. Kidneys with a filtration rate lower than 15 ml/min were considered incurable and were primarily switched to RNU.

The Paris System of Reporting Urinary Cytology was used to describe cytological findings before and 4 weeks after the surgery. Afterwards, it was a standard procedure at follow‐up visits. All cases underwent cystoscopy and ureteroscopy with UTUC mandatory primary tumour biopsy before the surgery; suspicious bladder or UT lesions were biopsied to exclude concomitant or multifocal disease. Importantly, the biopsy was performed as a separate procedure, at least four weeks before the definitive surgery. A single installation of Mitomycin was used to prevent bladder recurrence after diagnostic procedures. Pathological evaluation was based on the WHO 2016 classification. Low‐grade cases were excluded from the final dataset.

### 2.5 Systemic treatment

Each case was discussed by a multidisciplinary board with experts in the field. Perioperative systemic chemotherapy (neoadjuvant or adjuvant) was the preferred treatment approach and was administered to all patients included in the final analysis. A standard volume was a general number of 4 cycles (Gem‐Cis) which could have been done pre ‐ or postoperatively or split between these modalities. Indications, as well as contraindications, were stated depending on the general status, kidney function, concomitant pathology deterioration and level of complication according to CTCAE version 5.

### 2.6 Surgical technique

All surgical interventions were performed using open access. Primarily ureter was mobilized, wall‐thickening margins were identified. Afterwards, ureter was ligated and crossed on both sides. For distal ureteral tumours ‐ orifice resection was performed. Margins from both sides were checked with a frozen section during the surgery. In cases of positive margin, further ureteral resection was done, until the healthy end was verified by the pathologist. Lymph Node dissection was performed only in cases of clinically positive signs on CT scans.

Type of reconstruction depended on the length of the removed part. For the distal ureter, only one type of reconstruction was used ‐ neostomy. The plasty of the proximal part was conditioned by the length of the involved ureteral tissue. In cases smaller than 4 cm, a self‐tissue reconstruction was used (end‐to‐end anastomosis for mid‐ureter and Andersen‐Heinz technique for upper‐third). When the length exceeded 4 cm ‐ ureteral‐ileal interposition was applied. Upper tract was drained with a double‐J stent after surgery, which was removed in a 14–28 day period after surgery, in case there were no obstructive complications. Complications were assessed using Clavien‐Dindo scoring during 90 days after readmission.

### 2.7 Follow‐up

Visits were planned every three months during the first 2 years after surgery, afterwards every 6 months. Every follow‐up check‐up included cystoscopy, CT‐urography and urine cytology. Ureteroscopic investigations were standardly performed at 3 months after surgery in the operation room to exclude early recurrence or residual disease; afterwards only in cases of clinical significance. Superficial bladder recurrences were managed endoscopically and handled in accordance with non‐muscle invasive bladder cancer guidelines. In cases of upper tract recurrence ‐ RNU was performed. Cases of invasive bladder recurrence or progressive disease further tactics were discussed during multidisciplinary boards.

### 2.8 Statistical analysis

Statistical significance was determined using the SPSS software version 24.0 (IBM United States Software Announcement 216–071 March 15, 2016). Demographic and clinical parameters of both groups were compared using the t‐test (within the normal distribution), Mann–Whitney U test (for non‐parametric values) and Chi‐squared test (with non‐verification correction). Survival was assessed using the Kaplan–Meier estimator with the log‐rank test. Univariable Cox regression models were further used to identify whether tumour location influenced recurrence‐free, progression‐free and overall survival. Two‐sided statistical significance was defined as p‐values<0.05.

## 3 RESULTS

Over a 4‐year recruitment period, a total of 41 patients were included in the final analysis—21 with tumours located in the proximal ureter and 20 with tumours in the distal ureter. These patients had complete and consistent clinical, pathological and follow‐up data. During the recruitment phase, 11 patients were excluded due to reasons such as withdrawal of consent, loss to follow‐up, incomplete clinical data or changes in treatment strategy and were therefore considered dropouts.

Groups comparison by the basic clinical parameters is shown in Table 1. Cohorts were comparable by age, sex, BMI, ECOG status, T‐stage, clinical regional lymph node enlargement, tumour length and preoperative eGFR, primary pathology and cytology (p > 0.05). A slightly higher number of patients with enlarged regional lymph nodes and higher eGFR was seen among cases with proximal location, which however was not statistically significant. Average affected kidney function was higher in the proximal location cohort ‐ 29 ± 12 vs 22 ± 6 ml/min (z‐score 2.15; p = 0,031). There were 10 patients (50%) which underwent upper tract drainage before surgery in the distal location group and 11 (52%) ‐ in the proximal location cohort. Neodjuvant chemotherapy was performed in 15 (75%) and 17(80%) cases respectively. Adjuvant chemotherapy was favoured among patients where neoadjuvant setting was not used.

**TABLE 1:** Comparison of basic clinical parameters between groups.

Indicator/group	Distal	Proximal	p value
	n = 20	n = 21	
Age, years	70 ± 5,5	67,8 ± 6,3	p = 0.72
Sex, m/w	4/16	5/16	p = 0.76
BMI, kg/m^2^	26,9 ± 3,4	27,7 ± 3,1	p = 0.63
ECOG	1[0,1]	1[0,1]	p = 0.58
T2	16	16	p = 0.76
T3	4	5	
cN0	12	17	p = 0.14
cN1	8	4	
Tumour length, mm	36 ± 9	38 ± 14	p = 0.46
preoperative eGFR, ml/min	61,3 ± 11,7	66,8 ± 15,3	p = 0.22
Primary Pathology			
No tumour Cells	3	2	p = 0.26
LG	2	4	
HG	15	15	
Primary Cytology			
Negative	1	5	p = 0.65
Suspicious HG	7	4	
HG	12	12	
Systemic therapy			
Neoadjuvant	15	17	p = 0.64
Adjuvant	5	4	

Locations were compared in terms of perioperative parameters (see Table 2). All the patients with distal ureteral tumour location after SU underwent neostomy reconstruction. In the proximal location ‐ 9 (43%) end‐to‐end anastomosis, 8 (38%) Andersen‐Heinz plasty and 4 (19%) ureter‐ileum interposition. No difference between groups was observed regarding preoperative haemoglobin, intraoperative blood loss and 90‐day postoperative complication rate. One patient with a distal UTUC location died after surgery due to heart failure. All short‐term complications were managed conservatively with no patients requiring repeated surgery.

**TABLE 2:** Comparison of perioperative parameters between groups.

Indicator/group	Distal	Proximal	p value
	n = 20	n = 21	
Preoperative Haemoglobin, mg/dL	12 ± 2,8	12,1 ± 3	p = 0.85
Surgical duration, min	129 ± 17	137 ± 24	p = 0.51
Average bloodloss, ml	258 ± 98	282 ± 141	p = 0.67
Any complication, n (%)	10 (50)	15 (71)	p = 0.16
Grade ≥3 complications, n (%)	3 (15)	3 (14)	p = 0.94
Postoperative stay, days	7,6 ± 2,9	8,7 ± 3,8	p = 0.51
30‐day readmission rate, n(%)	2 (10)	3 (14)	p = 0.68

Final pathology confirmed high‐grade urothelial carcinoma in all analysed patients (n = 41). There were 5 patients in each group with locally advanced disease (pT3 or pN1 or pT3/pN1) ‐ 3/17 (pT3) and 4/16 (pN1) for distal location; 4/17 (pT3) and 3/18 (pN1) for proximal location. Long‐term follow‐up data and functional outcome revealed in Table 3. Recurrent upper tract obstruction was noticed in 3 (15%) and 4 (19%) cases in distal and proximal groups respectively. Two person in each group required permanent re‐stenting (due to hydronephrosis after DJ removal). One patient in each group underwent surgical reanastomosis and in one case in the group of proximal location due to high obstruction ureteral‐ileal interposition was performed (all three cases developed anastomosis strictures).

**TABLE 3:** Long‐term follow‐up (24 months) between groups.

Indicator/group	Distal	Proximal	p value
	n = 20	n = 21	
Recurrent obstruction, n (%)	3 (15)	4 (19)	p = 0.73
postoperative eGFR, ml/min	60,4 ± 8,5	59,4 ± 11,4	p = 0.81
Local Recurrence, n (%)	3 (15)	6 (28)	p = 0.29
Bladder Recurrence, n (%)	4 (20)	3 (14)	p = 0.62
Disease Progression, n (%)	3 (15)	5 (23)	p = 0.47

**FIGURE 1:**
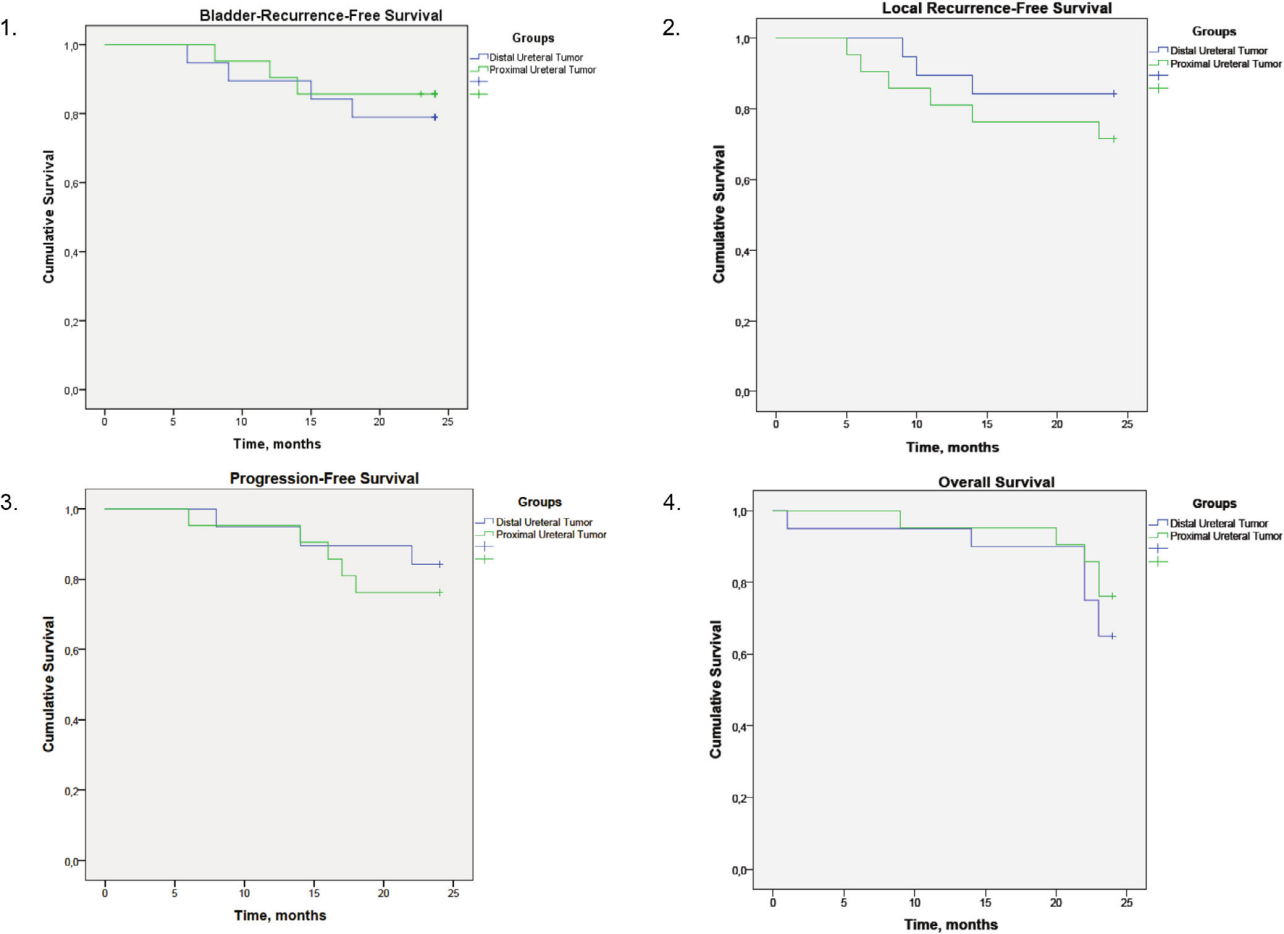
Groups comparison by local recurrence‐free (1), bladder‐recurrence‐free (2), progression‐free (3) and overall survival (4).

All patients were followed up for a minimum of 24 months. The last follow‐up was conducted in March 2024. The median follow‐up period for the entire cohort was 36 months (range: 24–60 months). Local recurrence‐free survival, Urinary Tract Recurrence‐free survival, Progression‐free survival and Overall Survival curves are shown in Figure 1. Table 3 presents numerical data on patients with local recurrence, bladder recurrence and disease progression. No significant differences were found between patients with proximal versus distal ureteral tumours in terms of 2‐years OS (65% vs. 77%; p = 0.43). All patients in both groups that experienced local recurrence underwent secondary RNU (3 (15%) vs 6 (28%) in the distal and proximal ureteral locations respectively).

The Kaplan–Meier curve presents data up to 24 months to reflect uniform follow‐up for the entire cohort and avoid bias from censored data in later periods. A separate analysis of long‐term outcomes beyond 24 months is planned and will be reported in future work.

## 4 DISCUSSION

Upper tract urothelial cancer is a highly aggressive disease which often requires a combined treatment approach. Surgical interventions to treat UTUC have been for decades limited to RNU with bladder cuff excision, which provides perfect local control.^3^ The same situation was observed in terms of systemic therapy, where platinum‐based regimens were for long considered a gold standard for advanced urothelial cancer.^2^ Further investigation of these two treatment modalities however revealed another problem ‐ high incidence of chronic kidney insufficiency among patients with UTUC. This results in a low level of chemotherapy prescription after the surgery, high levels of incomplete dosing and therefore decreased cancer‐specific survival in invasive UTUC.^3^

There were found two potentially effective ways to escape the kidney insufficiency trap ‐ neoadjuvant chemotherapy and kidney‐sparing surgery. The first one aims to catch the moment before impaired, however, a functioning kidney is removed, thus being applied to the patient with a relatively preserved general filtration rate.^4^ This led to sufficient findings, revealing a significantly prolonged disease‐specific survival within the cohort that receives presurgical systemic treatment.^15^ The other major findings of these studies are a 53–76% objective response rate, 11–14% of complete responses and up to 60% of pathological downstaging. Taking to account this data, some patients might be undergoing radical surgery, where the renal unit can be potentially saved by a kidney‐sparing approach. It was also shown that Neoadjuvant gemcitabine/cisplatin chemotherapy in invasive UTUC enables significant tumour regression, allowing for a safe organ‐sparing approach in selected patients, with better postoperative kidney function and improved 2‐year recurrence‐free survival compared to radical nephroureterectomy.^16^

Endoscopic procedures have already proved their high efficacy and compatible survival results for low‐risk UTUC, which however can not be applied to high‐risk disease. SU could be performed in these cases, however it is only recommended for distal ureteral lesions.^12^ Tumours of its proximal part are widely considered to be more surgically complex to reconstruct, which strongly limits organ‐sparing management in this area. Despite this, approaches towards organ‐sparing management of this zone were also investigated, showing relatively good outcomes on relatively small retrospective cohorts of patients.^14^, ^17^, ^18^ In fact, prognosis often depends on the tumour biology, whereas for highly aggressive disease response to systemic treatment is more crucial than surgery type.^19^, ^20^, ^21^, ^22^, ^23^

In the present study, we have performed a prospective non‐randomized cohort study which aimed to compare safety and oncological outcomes of the patients with proximal and distal lesion locations in the ureter.

This study provides a comparative analysis of patients undergoing surgery for upper tract urothelial carcinoma (UTUC) with tumours located in either the distal or proximal ureter. Despite similarities in baseline characteristics between groups, differences in functional outcomes and surgical approaches highlight important considerations for clinical decision‐making.

Patients in the distal and proximal cohorts were comparable in terms of age, sex, BMI, ECOG status, T‐stage, tumour length, clinical lymph node status, preoperative estimated glomerular filtration rate (eGFR) and pathological findings (p > 0.05). Notably, the proximal cohort had slightly higher preoperative kidney function, as evidenced by the average affected kidney eGFR (29 ± 12 vs. 22 ± 6 ml/min; p = 0.031), potentially reflecting the differential impact of tumour location on renal drainage and function. However, the clinical significance of this finding warrants further investigation.

Perioperative outcomes demonstrated distinct surgical approaches depending on tumour location. Neostomy reconstruction was universally employed for distal tumours, while proximal cases required more diverse techniques, including end‐to‐end anastomosis (43%), Andersen‐Heinz plasty (38%) and ureter‐ileum interposition (19%). These differences underscore the technical complexity of managing proximal ureteral tumours, necessitating individualized surgical planning based on tumour location and patient‐specific anatomy.

While intraoperative and postoperative complications were comparable between groups, the single mortality in the distal cohort due to heart failure highlights the need for careful perioperative assessment and optimization of high‐risk patients. All other complications were managed conservatively, and no reoperations were required, reflecting the overall safety and feasibility of the surgical approaches utilized.

Oncological outcomes, including recurrence‐free survival (RFS), progression‐free survival (PFS) and overall survival (OS), were comparable between groups, suggesting that tumour location does not significantly influence long‐term oncological prognosis. Here we should notice that the risk of local recurrence was relatively high between groups (15 vs 28%) and should have been secondary managed by RNU. Recurrent upper tract obstruction was observed in both cohorts (15% in distal vs. 19% in proximal). While most cases were managed with stenting or surgical reanastomosis, the requirement for ureteral‐ileal interposition in one proximal case highlights the potential for more complex postoperative challenges in this subgroup.

This study reinforces the need for tailored approaches in managing UTUC based on tumour location. The findings highlight that proximal ureteral tumours may demand more intricate surgical interventions and present with higher preoperative kidney function, whereas distal tumours are more likely to be managed with standardized techniques. Comparable oncological outcomes suggest that location does not compromise the effectiveness of surgical treatment, emphasizing the importance of individualized treatment planning to optimize functional and oncological outcomes.

Future studies with larger cohorts and longer follow‐up periods are needed to further elucidate the impact of tumour location on postoperative renal function, quality of life and survival outcomes.

The Oncological field is dynamically changing. New systemic agents appear opening the way to new treatment modalities and personalized therapeutic pathways. Perhaps prolonged survival received on different combinations of immune checkpoint inhibitors may state an important question: should we remove the kidney in responders or should we perform primary surgical treatment at all. With current investigational data, we hypothesize that there exist no functional or oncological differences in outcomes regardless of the ureteral tumour location. The kidney can be preserved in cases when the function is higher than 15 ml/min and the surgical centre has enough surgical experience to perform self‐tissue or ileal reconstruction. The extent of the primary investigation should carefully provide information about the length of the involved ureter, the presence of multifocality, concomitant bladder cancer and kidney function for more precise decision‐making. These patients should receive platinum‐based chemotherapy in neoadjuvant/adjuvant settings to facilitate better local and systemic oncologic control.

This study has several limitations that should be considered. First, it was conducted as a single‐centre analysis, which may limit the external validity and generalizability of the findings. Second, the sample size was relatively small, which may reduce the statistical power and the ability to detect associations. The inclusion of patients with locally advanced diseases introduces heterogeneity that may impact the interpretation of outcomes. Additionally, some patients underwent adjuvant chemotherapy, which could have influenced survival and recurrence data, acting as a potential confounder. It is important to note that in the context of upper tract urothelial carcinoma, assembling a sufficiently large and homogeneous patient cohort is inherently challenging due to the rarity of the disease. As a result, completely eliminating such biases from the final analysis is difficult. Future multicenter studies with larger, well‐defined cohorts are necessary to validate these findings and further clarify the prognostic and therapeutic implications in UTUC.

## 5 CONCLUSION

**Segmental ureterectomy** with further reconstruction can be safely performed regardless of the tumour location. Kidney sparing‐surgery at both proximal and distal parts of the ureter have shown equal oncological and functional outcomes. Risk of ureteral stenosis in the current study was not plasty type dependent.

## AUTHOR CONTRIBUTIONS

*Conceptualization*: Maksym Pikul, Prokip Gordiychuk, Eduard Stakhovsky. *Data Curation*: Maksym Pikul. *Formal Analysis*: Maksym Pikul. *Supervision*: Prokip Gordiychuk, Eduard Stakhovsky. *Writing – Original Draft Preparation*: Maksym Pikul. *Writing – Review and Editing*: Prokip Gordiychuk, Eduard Stakhovsky.

## CONFLICT OF INTEREST STATEMENT

There are no conflict of interests to declare.

## DISCLOSURE OF THE USE OF GENERATIVE AI AND AI‐ASSISTED TECHNOLOGIES

The authors did not use generative AI or AI‐assisted technologies in the process of medical writing or statistical analysis.

## DISCLOSURE

The study was approved by the Institutional Review Board and the local ethics committee (local ethics committee agreement No. 134, Kyiv, 26 March 2019) and was conducted according to the Declaration of Helsinki and the Good Clinical Practice guidelines. The databases used in the study are the intellectual property of the National Cancer Institute of Ukraine. A special informed consent was designed for the study and signed by all the patients in the presence of the urologists involved in the investigation.

## Data Availability

The data sets generated during and/or analysed during the current study are available from the corresponding author on reasonable request.
